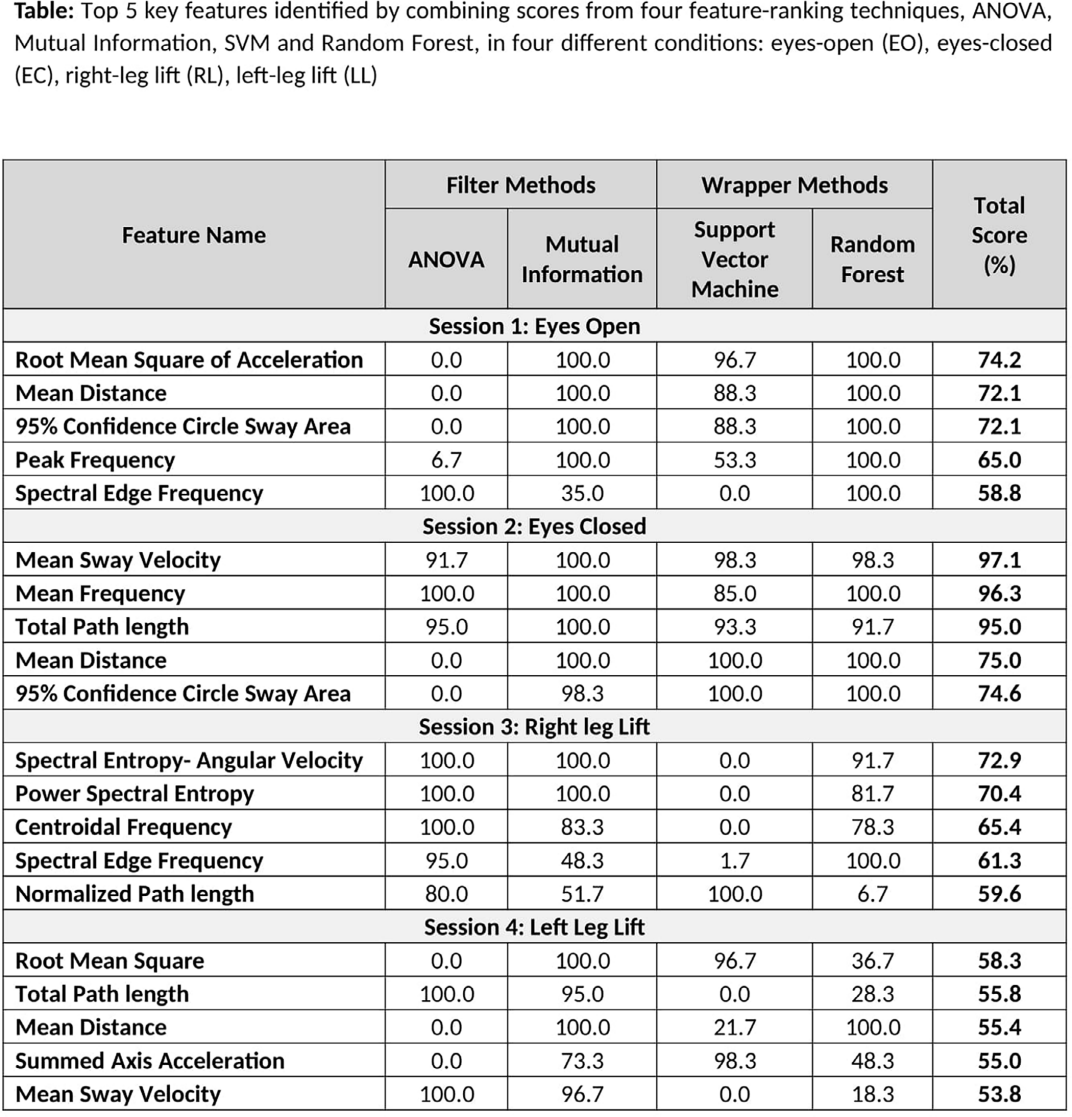# Identification of Balance biomarkers using Inertial sensor for Early Detection of Dementia

**DOI:** 10.1002/alz.088240

**Published:** 2025-01-09

**Authors:** Mobeena Jamshed, Ahsan Shahzad, Kiseon Kim

**Affiliations:** ^1^ National University of Sciences and Technology (NUST), Islamabad, ICT Pakistan; ^2^ School of Electrical Engineering and Computer Science Gwangju Institute of Science and Technology, Gwangju 61005, Gwangju Korea, Republic of (South)

## Abstract

**Background:**

Early‐stage dementia, Mild Cognitive Impairment (MCI), is challenging to diagnose since it is a transient condition distinct from complete cognitive collapse. Recent clinical research studies have identified that balance impairments can be a significant indicator for predicting dementia in older adults. Accordingly, we aimed to identify key balance biomarkers using wearable inertial sensors for early detection of dementia/MCI.

**Method:**

At National Research Center for Dementia (South Korea), 60 participants (30 Cognitively Normal‐CN, 30 Mild Cognitive Impairment‐MCI) were selected based on assessments conducted by medical professionals at Chosun University Hospital / Chonnam National University Hospital (Gwangju). Shimmer‐3 inertial sensor was placed on lower back of participants, with a tri‐axial accelerometer and tri‐axial gyroscope. The data was collected under four different conditions: eyes‐open (EO), eyes‐closed (EC), right‐leg lift (RL), left‐leg lift (LL). A set of 76 postural sway measures were considered with 43 time‐domain and 33 frequency‐domain features.

Feature ranking was executed through the utilization of "Leave‐One‐Subject‐Out (LOSO)" cross‐validation technique for each condition. Filter methods: one‐way‐ANOVA (Analysis of Variance), Mutual Information and Wrapper methods: Random Forest, Support Vector Machine (SVM) were utilized to evaluate feature subsets. The proportional value of each feature was calculated by the number of times it showed in the top feature list. The final score was obtained by combining scores from all the techniques, which indicated the relative weight of characteristics across the various approaches. Further feature analysis was conducted using SHAP (Shapley‐Additive‐exPlanations).

**Result:**

The multi‐step methodology, for feature selection, adopted in our research ensured a rigorous process, combining the strengths of Filter and Wrapper methods. In time‐domain: root‐mean‐square, mean‐distance, 95%‐confidence circle sway‐area, path‐length, mean sway‐velocity were the key features identified. While in frequency‐domain: spectral edge‐frequency, mean‐frequency, peak‐frequency, centroid‐frequency, spectral‐entropy were found to be the principal features of relevance. These features were also included in the top list of features based on SHAP impact value, hence proving the effectiveness of our method in identifying important biomarkers for detection of MCI.

**Conclusion:**

The inertial sensors based postural balance assessment provides useful biomarkers that can facilitate early screening of MCI / dementia in home settings.